# Symmetric Off‐Axis Spectral Encoding Enables High‐Throughput Six‐Color Optical Sectioning Imaging

**DOI:** 10.1002/nap2.70367

**Published:** 2026-11-07

**Authors:** Jiangjiang Zhao, Zhangheng Ding, Zhan Zhang, Jie Zhang, Shuhong Qi, Jing Yuan

**Affiliations:** ^1^ MoE Key Laboratory for Biomedical Photonics Wuhan National Laboratory for Optoelectronics Huazhong University of Science and Technology Wuhan China

**Keywords:** fluorescence microscopy, high‐throughput imaging, multicolor imaging, off‐axis detection, optical sectioning

## Abstract

Multicolor fluorescence microscopy is central to biological imaging, but simultaneous detection of additional colors typically reduces throughput, increases spectral crosstalk, or lowers signal‐to‐noise ratio. We developed symmetric off‐axis spectral encoding microscopy (sym‐OSEM), which uses paired spatial encoding and differential decoding to separate fluorescence channels and suppress common‐mode out‐of‐focus background. The differential decoding requires no specimen‐specific reference spectrum, point spread function, or training data. All fluorophores are excited and detected simultaneously, so acquisition time is largely independent of channel count within the demonstrated two‐ to six‐color configurations. Simulations and experiments using cell preparations, a serially imaged 300 μm‐thick mouse‐brain volume, and lymph node sections validated the approach. Four‐color imaging of U2OS cells covered 6.362 mm^2^ min^−1^, 59‐fold faster than a commercial confocal microscope under the tested acquisition settings, while retaining comparable image quality. Simultaneous six‐color imaging resolved the distributions of immune‐cell and stromal markers in lymph‐node tissue. Sym‐OSEM therefore provides a strategy for combining multicolor separation, optical sectioning, and high‐throughput acquisition in a single imaging framework.

## Graphical abstract

Within an off‐axis imaging framework, symmetric spatial encoding enables differential separation of fluorescence components excited at different wavelengths while suppressing common‐mode out‐of‐focus background. Combined with parallel emission‐band detection and residual crosstalk correction, symmetric off‐axis spectral encoding microscopy (sym‐OSEM) enables high‐throughput, simultaneous six‐color optical sectioning imaging of lymph node sections.

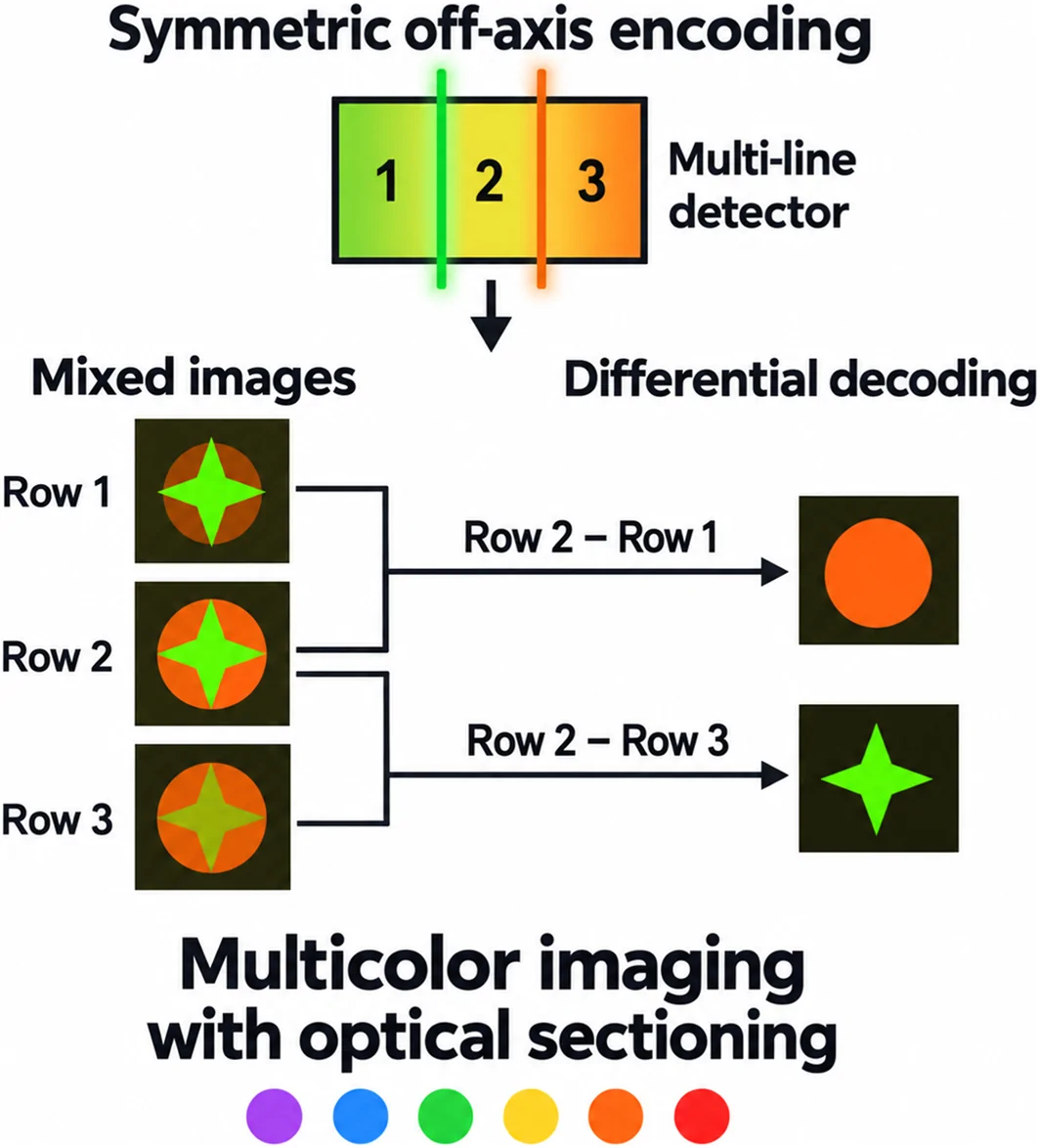

## 1 Introduction

Multicolor fluorescence microscopy enables examination of multiple molecular targets within the same specimen and is therefore important for resolving biological organization and function [1, 2, 3]. Its multiplexing capacity is constrained by the broad excitation and emission spectra of common fluorophores and the limited accessible spectral window [4, 5, 6]. Consequently, spectral overlap and crosstalk increase as more fluorophores are detected simultaneously.

Sequential imaging mitigates spectral crosstalk by exciting and detecting fluorophores individually through wavelength‐selective filters [7, 8, 9, 10]. However, sequential acquisition sacrifices temporal correspondence and throughput, while narrowband filtering discards emitted photons and can reduce signal‐to‐noise ratio (SNR). Parallel detection systems use dispersive or dichroic elements to distribute fluorescence among several detectors [11, 12, 13, 14, 15], but their complexity and detector requirements increase with channel count. A practical method that combines simultaneous acquisition, low crosstalk, high photon efficiency, and optical sectioning for four or more colors remains difficult to achieve.

We previously developed off‐axis detection as a modulation strategy for line‐scanning microscopy [16, 17, 18, 19, 20]. Subdetectors conjugated to different regions of the illumination point spread function (PSF) record distinct spatial modulations. We subsequently introduced off‐axis spectral encoding microscopy (OSEM), in which wavelength‐dependent lateral offsets encode fluorophores through different subdetector mixing ratios [21]. In the original OSEM implementation, PSF‐based linear decoding was effective only for specimens approximately 1–2 μm thick, whereas thicker specimens required a trained deep‐learning model. The decoding accuracy and computational cost therefore depended on measured or learned priors. These limitations arise because the original encoding matrix becomes poorly conditioned when spectral overlap and out‐of‐focus background increase.

Here, we introduce symmetric off‐axis spectral encoding microscopy (sym‐OSEM), which redesigns the encoding geometry to enable reference‐free differential decoding. Subdetectors placed symmetrically about an illumination line record matched modulation for one fluorophore but different off‐axis modulation for the adjacent fluorophore. Subtracting the paired measurements cancels the matched spectral component and the approximately common‐mode out‐of‐focus background, while preserving the differentially modulated in‐focus signal. This deterministic operation requires no specimen‐specific PSF, reference spectrum, or training data for decoding.

Sym‐OSEM provides three linked capabilities. First, symmetric encoding replaces prior‐dependent unmixing with a direct differential calculation. Second, parallel excitation and detection preserve acquisition speed across the demonstrated two‐ to six‐color configurations, although additional colors require corresponding detection capacity. Third, the same subtraction used for color separation suppresses common‐mode out‐of‐focus background and thereby provides optical sectioning. A three‐camera splitter extends the two‐color‐per‐camera design to six simultaneous channels. We evaluated the method in simulation and in two‐color cellular and brain‐volume imaging, four‐color cellular imaging, and six‐color lymph node imaging, comparing it with PSF‐based linear decoding, deep‐learning decoding, sequential reference imaging, and commercial confocal microscopy.

## 2 Methods

### 2.1 Symmetric Off‐Axis Spectral Encoding

The sym‐OSEM system combines symmetric off‐axis encoding, differential decoding, and parallel three‐camera detection for simultaneous six‐color optical sectioning (Figure 1a). Four lasers (405‐06‐01‐0100, 488‐06‐01‐0100, 561‐06‐01‐0100, and 633‐06‐01‐0080; Cobolt) provide the commonly used visible excitation wavelengths. Three dichroic mirrors (DM1, ZT568rdc‐25 × 36; DM2, ZT502rdc‐25 × 36; DM3, ZT442rdc‐25 × 36; Chroma) combine the beams. Lenses L1 (*f* = 7.5 mm, AC050‐008‐A‐ML, Thorlabs) and L2 (*f* = 100 mm, AC254‐100‐A, Thorlabs) expand the combined beam. A cylindrical lens (CL, ACY254‐100‐A, Thorlabs) focuses the expanded beam into a line. This line is then projected onto the objective focal plane by an illumination tube lens (TL1, AC254‐150‐A‐ML, Thorlabs) and either a 20×, 1.0‐numerical aperture (NA) water‐immersion objective (XLUMPLFLN20XW, Olympus) or a 60×, 1.1‐NA water‐immersion objective (LUMFLN60XW, Olympus). The line‐scanning geometry acquires an entire detector row during each exposure.

**FIGURE 1:**
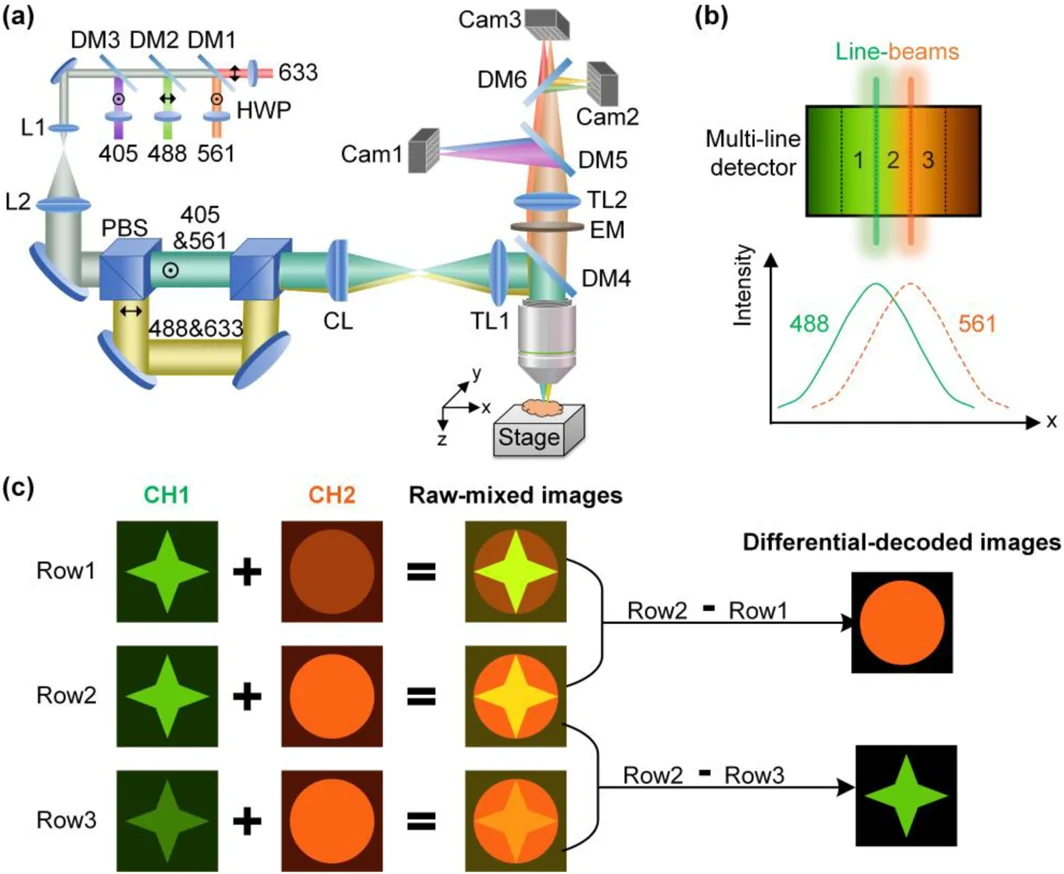
Principle of symmetric off‐axis spectral encoding multicolor imaging. (a) Schematic of the imaging setup. (b) The line‐illumination‐beam arrangement uses the two fluorescence channels detected by camera 2 as an example. (c) An elementary three‐row illustration of paired differential decoding, also using the two fluorescence channels of camera 2 as an example.

The same objective collects fluorescence and passes it through dichroic mirror DM4 (ZT405/488/561/640rpcv2, Chroma) and emission filter EM (ZET405/488/561/640mv2, Chroma) to reject excitation light. Two dichroic mirrors (DM5, FF520‐Di02‐25 × 36; DM6, FF593‐Di03‐25 × 36; Semrock) divide the emission into three spectral groups, which are projected through detection tube lens TL2 (U‐TLU, Olympus) onto three sCMOS cameras (Cam1‐Cam3, ORCA‐Flash 4.0, Hamamatsu). Cam1 records the 405 nm‐excited fluorescence and the < 520 nm component of the 488 nm‐excited fluorescence. Cam2 records the > 520 nm component of the 488 nm‐excited fluorescence and the < 593 nm component of the 561 nm‐excited fluorescence. Cam3 records the > 593 nm component of the 561 nm‐excited fluorescence and the 633 nm‐excited fluorescence. Each camera therefore records two encoded channels, and the three cameras operate in parallel. A three‐axis translation stage (*x*, ABL20020; *y*, ANT130; *z*, AVL125; Aerotech) enables large‐area and volumetric scanning.

Symmetric encoding requires positioning each excitation line at a designated seam between adjacent subdetector rows. For example, for the 488 and 561 nm excited channels recorded by Cam2 (Figure 1b), the 488 nm line is aligned with the seam between Rows 1 and 2, and the 561 nm line with the seam between Rows 2 and 3. After expansion, beam positions are adjusted to achieve sub‐pixel control. A half‐wave plate (AHWP05M‐580, Thorlabs) in each laser path sets the polarization state (S‐polarization for 405 and 561 nm; P‐polarization for 488 and 633 nm). A polarizing beam splitter (CCM1‐PBS251/M, Thorlabs) separates the combined beams into two paths, and the angles of mirrors (PF10‐03‐P01, Thorlabs) mounted in precision kinematic mounts (KS2D, Thorlabs) are adjusted to control their lateral positions at the detector plane. During system alignment, each excitation line images a wavelength‐matched fluorescent reference plate (FSK5, Thorlabs), and we align its center relative to the camera rows. We perform this alignment during system setup rather than for each specimen. We mount the optical system on a marble vibration‐isolation platform to reduce positional drift.

We illustrate the encoding principles using Cam2 as an example, as shown in Figure 1c. The four‐pointed stars and circles represent two fluorophores excited at 488 and 561 nm, respectively. The three rows correspond to subdetector images in Figure 1b. Because each excitation beam is offset to a different seam, the two fluorescence signals exhibit distinct intensity distributions across the three subdetectors. The raw mixed image captured by the *i*th subdetector is a linear superposition of both fluorescence signals:

(1) $$I_{i}\left(x,y,z\right)=\sum_{j=1}^{n}p_{j}\left[H_{i,j}⊗X_{j}\right]\left(x,y,z\right)+B_{i}\left(x,y,z\right)+\eta_{i}\left(x,y,z\right),$$

where *n* is the number of fluorescence channels recorded by one camera (*n* = 2 here), *P_j_* is an intensity factor for fluorophore *j*, *H*_*i,j*_ denotes the PSF for excitation wavelength *j* at subdetector *i*, *X*_*j*_ is the distribution of the *j*th fluorophore, ⊗ denotes three‐dimensional convolution. *B*_*i*_ is a deterministic additive background with nonzero mean, such as dark current, baseline offset, or ambient stray light, and *ηᵢ* denotes a zero‐mean, signal‐dependent noise component, including shot noise and read noise. The scan‐coordinate derivation and the six‐fluorophore extension are provided in Supporting Information S1: Section 4.

### 2.2 Differential Decoding

The decoding algorithm exploits a simple but powerful symmetry. In a multicolor line‐scanning imaging system, the PSF distributions of two subdetectors symmetric about the *q*th line‐illumination beam are mirror‐symmetric:

(2) $$H_{-d,q}\left(x,y,z\right)=H_{+d,q}\left(-x,y,z\right).$$

Mirror symmetry motivates approximate response matching but does not guarantee equal responses for arbitrary specimens. Fixed subpixel registration of the paired responses in a common coordinate system reduces displacement. Let *H̃*_*i,q*_ denote the registered responses of the *i*th subdetector about the *q*th excitation line, given by

(3) $${\tilde{H}}_{i,q}=T_{i}H_{i,q},$$

where *T*_*i*_ is a fixed lateral translation. This correction is specimen‐independent and forms part of the one‐time calibration on the imaging instrument side. Any residual mismatch between the two symmetric subdetectors can be written as

(4) $$\varepsilon_{q}={\tilde{H}}_{+d,q}-{\tilde{H}}_{-d,q}.$$

To recover the monochromatic image of the *j*th excitation wavelength while canceling the *q*th excitation component, we subtract the registered row measurements from subdetectors *a* and *b*, symmetrically positioned about the *q*th line‐illumination spot:

(5) $$D_{j}={\tilde{I}}_{a}-{\tilde{I}}_{b}=P_{j}X_{j}⊗K_{j}+P_{q}X_{q}⊗\varepsilon_{q}+\Delta \tilde{B}+\Delta \tilde{\eta },$$

where $K_{j}={\tilde{H}}_{a,j}-{\tilde{H}}_{b,j},\Delta \tilde{B}={\tilde{B}}_{a}-{\tilde{B}}_{b},\Delta \tilde{\eta }={\tilde{\eta }}_{a}-{\tilde{\eta }}_{b}.$ When the paired responses for the unwanted component are matched ($\varepsilon_{q}=0$), and the additive bias is balanced ($\Delta \tilde{B}=0$), the subtraction yields a clean fluorescence signal of the *j*th excitation wavelength. The conditional mean of the differential decoding result satisfies:

(6) $$E\left[D_{j}|X\right]=P_{j}X_{j}⊗K_{j}.$$

Defocus weakens the spatial modulation *K*_*j*_ induced by line illumination, making the out‐of‐focus background approximately common across subdetectors. Differential decoding exploits this common‐mode property to suppress the out‐of‐focus contribution. Under ideal symmetry, differential decoding therefore removes the qth excitation component and the out‐of‐focus background simultaneously, yielding a clean, optically sectioned monochromatic image at the *j*th excitation wavelength.

For example, as shown in Figure 1c, to decode the 561 nm channel (orange circles), we subtract Row1 from Row2. The 488 nm signal (green stars) is identical in both rows and therefore cancels out. The out‐of‐focus background cancels as well. Only the 561 nm signal remains, with a clean optical sectioning effect. Similarly, to decode the 488 nm channel, we subtract Row3 from Row2. The 561 nm signal cancels, and the 488 nm signal is recovered.

Differential decoding relies on two conditions. First, the unwanted channel produces closely matched PSFs on the symmetric subdetectors, while the target channel produces a sufficiently large differential signal. The beam‐alignment procedure establishes this geometry. Second, the out‐of‐focus components are approximately common across subdetectors, while the in‐focus target remains spatially modulated. The imaging system’s inherent attenuation of high‐frequency information meets this condition.

In practice, line‐spot drift, subdetector misregistration, channel‐intensity imbalance, detector noise, gain mismatch, spherical aberration, and defocus can all introduce symmetry deviations and degrade decoding accuracy. We therefore evaluated differential decoding sensitivity to these error terms in simulation. We varied one error term at a time while keeping the remaining parameters at their nominal values. For each condition, we calculated the crosstalk increase, the broadening of the axial‐sectioning response, and the decoded image SNR.

In a joint‐error scenario in which all error terms were simultaneously set to half of their tolerance values, the crosstalk increase was 7.5%, axial‐response broadening was 1.1‐fold, and peak SNR was 26.1 dB. These results quantify the sensitivity of differential decoding to the considered error terms and show that, under the joint‐error condition, the performance degradation remains within the tolerance criteria used in this analysis (crosstalk increase < 10%, axial broadening < 2‐fold, SNR > 20 dB). Detailed results of the error simulation analysis are provided in Supporting Information S1: Section 1.

Sym‐OSEM differs from conventional spectral unmixing by encoding a matched component that can be removed through direct subtraction. Decoding therefore requires no specimen‐specific measured PSF, reference spectrum, or trained model; it relies only on a one‐time geometric alignment of the illumination lines and intensity balance between paired channels. The arithmetic operation is sufficiently simple for real‐time implementation.

## 3 Results

### 3.1 Simulation Validation and Performance Measurements

To theoretically validate the feasibility of symmetric off‐axis spectral encoding multicolor imaging, we constructed a dual‐color virtual sample model comprising randomly distributed spheres and tetrahedra in 3D space (Figure 2a). The virtual sample measured 40 μm × 40 μm × 20 μm, with spheres and tetrahedra representing two distinct fluorescent signals characterized by different excitation and emission spectra. For simulation experiments, we placed the imaging focal plane at the sample’s central layer (*z* = 10 μm). Using parameters of the 20×, 1.0‐NA water‐immersion objective at a 0.1 μm sampling rate, we calculated the 3D PSF distributions for subdetectors at various off‐axis positions. We simulated the sphere signal with 561 nm excitation and 590 nm emission, while the tetrahedron signal used 633 nm excitation and 660 nm emission. We generated monochromatic images for each subdetector in both channels by convolving each fluorescence signal in the sample model with its corresponding 3D PSF. We then summed the two monochromatic images from the same subdetector to obtain the corresponding encoded mixed images (Figure 2b). For each channel, we applied LiMo (Line Illumination Modulation) processing to the monochromatic images by subtracting the edge‐subdetector image from the central‐subdetector image. The resulting out‐of‐focus‐suppressed monochromatic sequential LiMo images served as ground truth.

**FIGURE 2:**
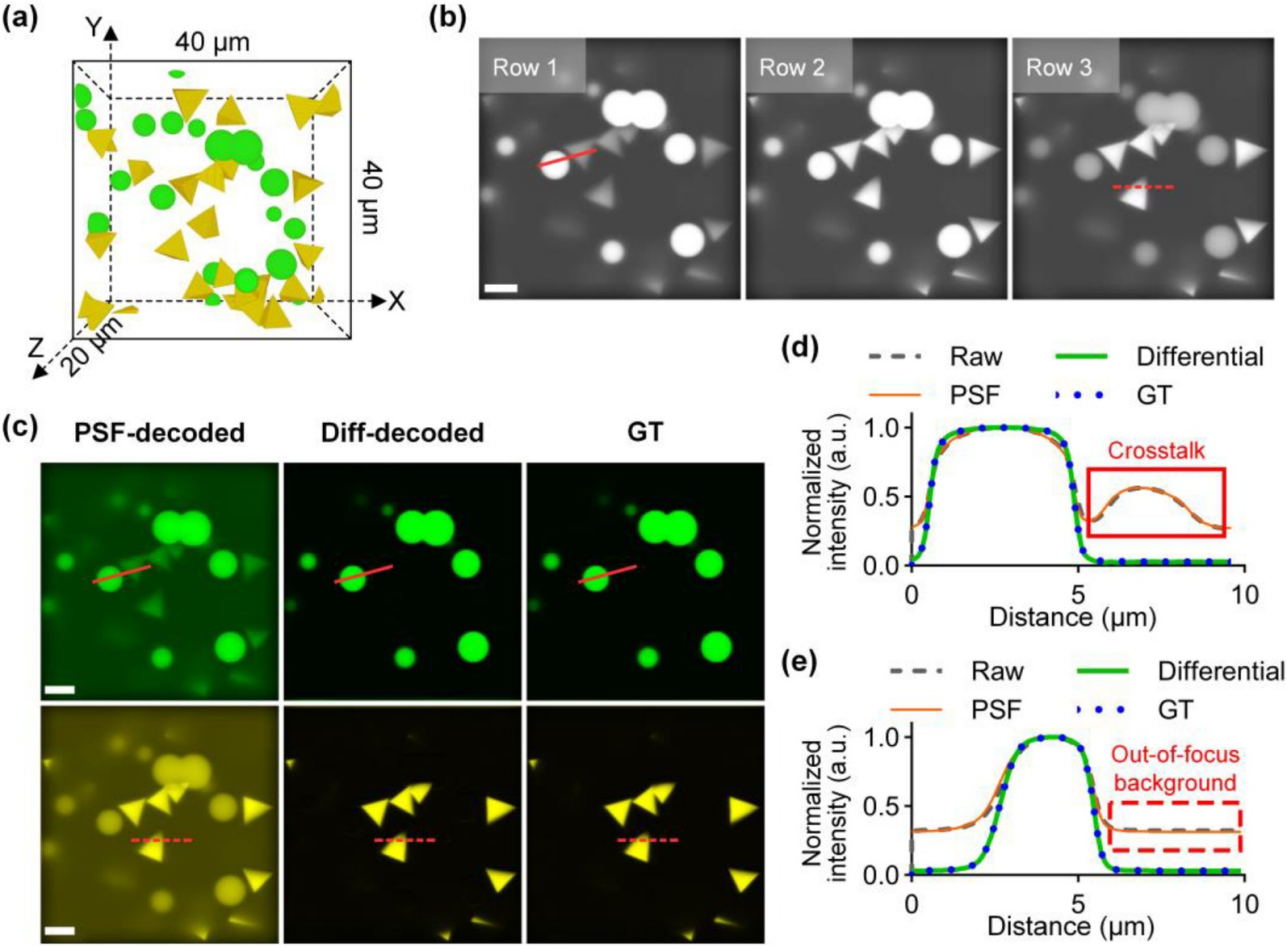
Simulated dual‐color imaging of a virtual sample. (a) Dual‐color virtual sample, with green spheres and yellow tetrahedra representing two distinct fluorescent signals. (b) Raw mixed images captured by subdetectors at different off‐axis positions. (c) Decoded monochromatic images (left to right: linear‐decoded, differentially decoded, and sequential LiMo reference images). (d) Signal intensity profiles along the red solid lines in the “Row1” image of (b) and in (c). (e) Signal intensity profiles along the red dashed lines in the “Row3” image of (b) and in (c). Scale bar: 5 μm.

We processed the raw mixed images using PSF‐based linear decoding and symmetric differential decoding (Figure 2c). Intensity profiles through the red solid lines in Figure 2b,c show residual tetrahedral signal after PSF decoding (Figure 2d), whereas differential decoding more closely reproduced the separately simulated channels. Profiles through the red dashed lines show that differential decoding also suppressed the simulated out‐of‐focus component (Figure 2e). These simulations validated the feasibility of sym‐OSEM under the assumed PSF symmetry and background model.

We then experimentally characterized the axial sectioning and three‐dimensional spatial responses. The axial sectioning response was measured using a fluorescent calibration slide (Argo‐SIM‐SLG034, Argolight) with 4 × 4 cross structures, in which horizontal lines were positioned at different axial planes (Supporting Information S1: Section 2). A 200‐plane *z*‐stack was acquired at 0.1 μm axial steps with the 60× objective. During acquisition, the sCMOS camera was operated in the 16‐row subarray mode to record images from different off‐axis subdetectors independently. Approximate wide‐field, line‐confocal, and differential‐decoded images were reconstructed from the same raw data. The axial response was quantified from the background‐subtracted integrated intensity of each horizontal line across the *z*‐stack. The full width at half maximum (FWHM) values of the axial responses were 3.74 ± 0.06 μm for the wide‐field image, 2.55 ± 0.05 μm for the line‐confocal image, and 1.84 ± 0.05 μm for the differential image (mean ± standard deviation, *n* = 16). Differential processing therefore narrowed the axial‐response width by approximately 50.8% relative to wide‐field imaging and by approximately 27.8% relative to line‐confocal imaging. These results should not be directly extrapolated to biological tissue imaging or other imaging conditions, such as different objective magnifications, as specimen scattering and response matching can affect sectioning performance.

We measured the three‐dimensional spatial response with 200 nm fluorescent beads (T14792, ThermoFisher) under three excitation/emission conditions: 488/515, 561/580, and 633/680 nm. We performed imaging with the same 60× objective, using 108‐nm lateral sampling and 100 nm axial steps. For each fluorescence channel, 15 randomly selected beads were analyzed along *X* (perpendicular to the illumination line), *Y*, and *Z*. Relative to the wide‐field image, line‐confocal imaging reduced the *X* and *Z* FWHM by approximately 23%–28% and 25%–30%, and differential decoding reduced them by approximately 38%–41% and 42%–45%, respectively. The *Y* response changed by less than 10%, consistent with the line‐scanning geometry, in which confocal sharpening and differential suppression act mainly perpendicular to the illumination line and along the axial direction. By subtracting adjacent subdetector images, the differential image further compressed the *X*‐direction PSF main lobe and canceled uniform out‐of‐focus background, yielding an additional reduction of approximately 13%–19% in *X* and 20%–26% in *Z* relative to line‐confocal (Supporting Information S1: Section 3).

### 3.2 Dual‐Color Imaging of BPAE Cells

To evaluate sym‐OSEM in a biological specimen, we performed single‐camera, two‐color imaging of a prepared bovine pulmonary artery endothelial cell (BPAE) slide (F36924, ThermoFisher) containing DAPI‐labeled nuclei and Alexa Fluor 488‐labeled actin filaments (F‐actin). The 405 and 488 nm lasers provided simultaneous excitation, and a six‐row sCMOS subarray recorded the mixed measurements using the 20× objective. We also acquired monochromatic images of the two fluorescence signals sequentially and processed them with the LiMo algorithm to provide reference images.

Figure 3a shows the raw mixed images captured by three adjacent off‐axis subdetectors (Rows 1 to 3, top to bottom). Magenta and green boxes highlight nuclear and F‐actin regions, respectively, with enlarged views in Figure 3b. As expected, all subdetector images contain both fluorescent signals but exhibit distinct intensity patterns. Specifically, the nuclear signals are strongest and nearly identical in Rows 1 and 2, but weaker in Row 3, because the 405 nm excitation beam is conjugate to the central seam between Rows 1 and 2. Similarly, the F‐actin signals are strongest and nearly identical in Rows 2 and 3, but weaker in Row 1, as the 488 nm excitation beam is conjugate to the seam between Rows 2 and 3.

**FIGURE 3:**
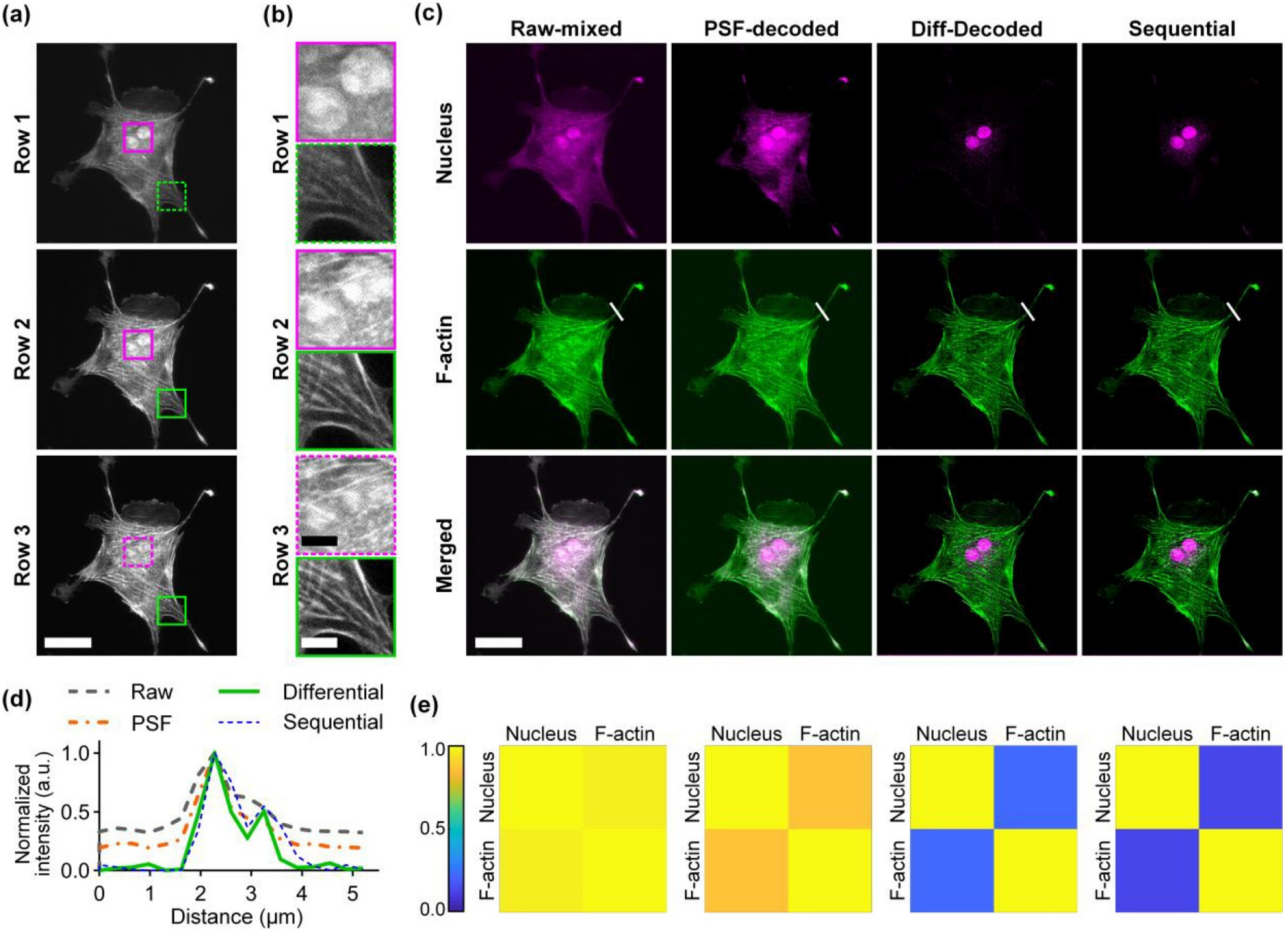
Dual‐color BPAE‐cell imaging with sym‐OSEM. (a) Raw mixed images recorded by three subdetectors. (b) Enlarged views of the boxed regions in panel (a). (c) Nuclear (magenta), F‐actin (green), and merged images. Columns show raw mixed, PSF linearly decoded, differentially decoded, and sequential reference images. (d) Intensity profiles along the white lines in panel (c). (e) Cross‐correlation‐coefficient heatmaps for the two recovered channels. Scale bars: 50 μm in panels (a) and (c), 10 μm in panel (b).

We applied both PSF‐based linear decoding and differential decoding to the mixed measurements. Figure 3c compares the raw mixed, PSF linearly decoded, differentially decoded, and sequential reference images. PSF decoding retained visible signal leakage, whereas differential decoding produced channel distributions closer to the sequential references. Intensity profiles through two adjacent actin filaments show that differential decoding sufficiently suppressed background to resolve both peaks (Figure 3d). Spatial cross‐correlation between the recovered channels was also reduced relative to the raw‐mixed and PSF‐decoded images (Figure 3e). Together, these measurements support simultaneous channel separation and optical sectioning in the prepared‐cell specimen.

### 3.3 Dual‐Color Volumetric Imaging of Mouse Brain Tissue

To demonstrate volumetric multicolor imaging capability, we performed single‐camera dual‐color imaging on a 300 μm‐thick mouse brain tissue block. We injected 100 nL of an adeno‐associated virus (AAV) vector encoding enhanced green fluorescent protein (eGFP) for sparse labeling into the primary motor cortex of a C57BL/6J mouse (#000664, Jackson Laboratory). After 3 weeks of viral expression, we harvested the brain and embedded the tissue in glycol methacrylate resin. During imaging, we immersed the sample in propidium iodide (PI) solution, yielding sparsely labeled eGFP‐expressing neurons and PI‐counterstained nuclei, and excited it with 488 and 561 nm lasers, respectively.

We used serial block‐face imaging with fluorescence micro‐optical sectioning tomography (fMOST), removing 1 μm from the tissue surface after each imaging plane. We acquired a 5 mm × 8 mm × 300 μm brain volume using a 20× objective at 0.325 μm × 0.325 μm × 1 μm voxel sampling in 3.8 h with real‐time differential decoding. Figure 4a shows the resulting two‐color data, with PI‐labeled nuclei displayed from the surface plane and eGFP‐labeled neurons shown as a maximum‐intensity projection through the serially acquired volume. Figure 4b,c show three‐dimensional reconstructions of the PI and eGFP channels within the boxed region.

**FIGURE 4:**
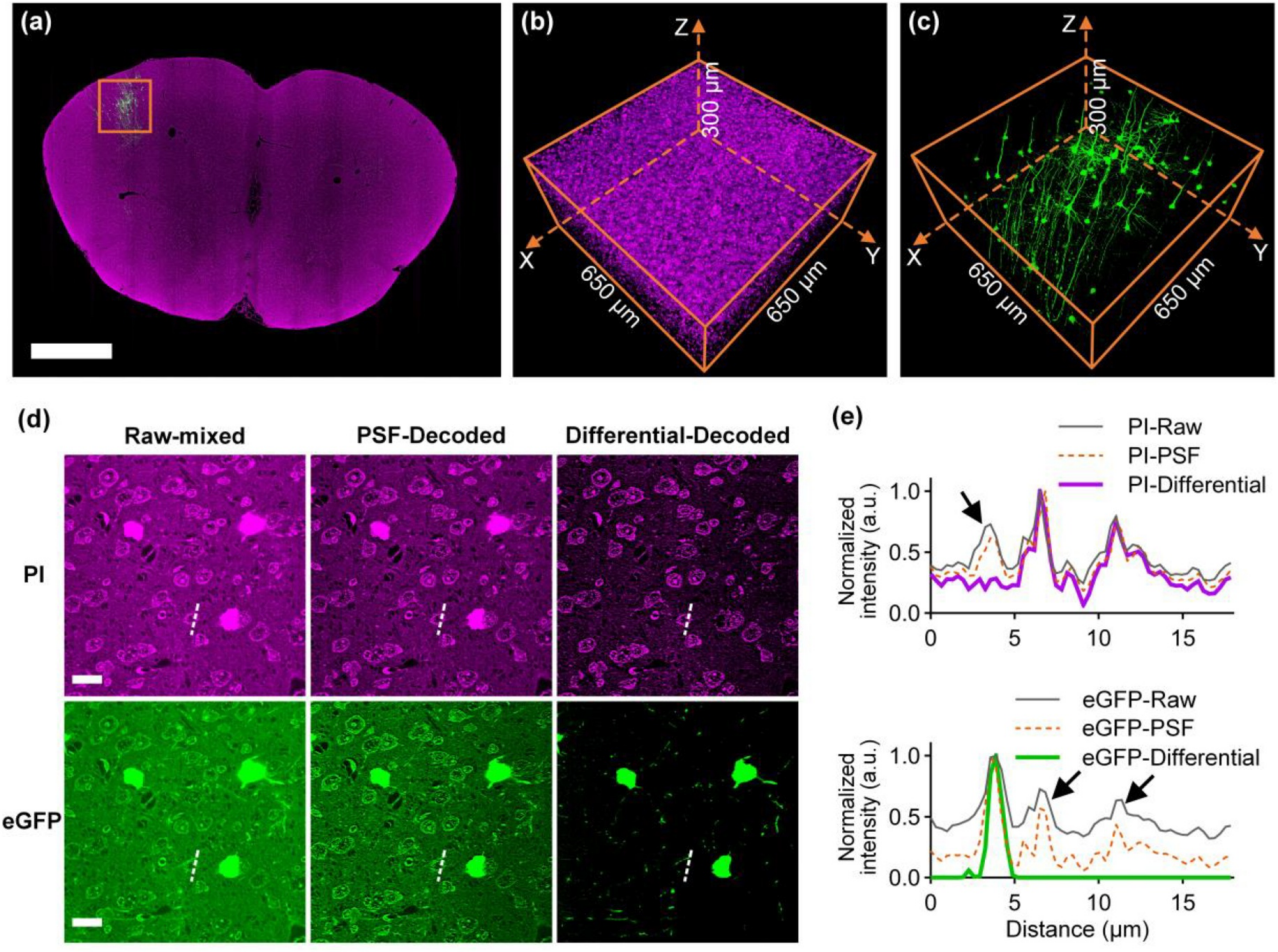
Dual‐color 3D imaging of a mouse brain tissue block. (a) Dual‐color merged image of nuclei and neurons in mouse brain tissue. Magenta represents PI‐labeled nuclei, shown as a single‐layer image at the sample surface; green represents eGFP‐labeled neurons, shown as a maximum‐intensity projection through the 300 μm‐thick sample. (b) 3D reconstruction of the PI signal in the boxed region of panel (a). (c) 3D reconstruction of the eGFP signal in the boxed region of panel (a). (d) Magnified views of the two fluorescence signals at the sample surface. From left to right: raw mixed, PSF‐linear‐decoded, and differentially decoded images. (e) Signal intensity profiles along the white lines in panel (d). Top: PI signal, bottom: eGFP signal. Scale bars: 1 mm in panel (a), 20 μm in panel (d).

Figure 4d compares raw mixed, PSF‐linear‐decoded, and differentially decoded surface‐plane images in a region containing both signals. Figure 4e shows the signal intensity profiles along the white lines in Figure 4d. Raw mixed and PSF‐decoded channels showed similar peak patterns, whereas differential decoding retained the nuclear peaks in the PI channel and the neuronal‐fiber peak in the eGFP channel. The reduced shared background further supports simultaneous channel separation and optical sectioning by differential decoding during serial block‐face volume acquisition. These results demonstrate that sym‐OSEM is compatible with serial block‐face sectioning and suitable for large‐volume imaging.

### 3.4 Four‐Color Imaging of U2OS Cells

To benchmark sym‐OSEM against an industry standard and demonstrate its throughput advantage, we simultaneously imaged four subcellular structures in U2OS cells. We labeled the nuclei (blue, Hoechst 33258), intermediate filaments (green, Alexa Fluor 488), microtubules (yellow, Alexa Fluor 594), and mitochondria (magenta, Alexa Fluor 647).

Figure 5a shows a four‐color sym‐OSEM image of a 1.483 mm × 2.052 mm region acquired with a 60×, 1.1‐NA water‐immersion objective in 28.7 s with real‐time differential decoding. We also imaged the 0.135 mm × 0.378 mm boxed region in Figure 5a using a commercial confocal microscope (LSM980, Zeiss) in Best Signal mode with a 63×, 1.4‐NA oil‐immersion objective and a similar acquisition time of 28.2 s (Figure 5b). Sym‐OSEM and LSM980 achieved area throughputs of 6.362 and 0.109 mm^2^ min^−1^, respectively, corresponding to an approximately 59‐fold advantage for sym‐OSEM with qualitatively comparable images. This advantage reflects the combined contributions of simultaneous multicolor acquisition enabled by symmetric off‐axis encoding and parallel line scanning. As the acquisition parameters were not fully matched between the two systems, the reported throughput ratio is specific to the settings summarized in Supporting Information S1: Table S2.

**FIGURE 5:**
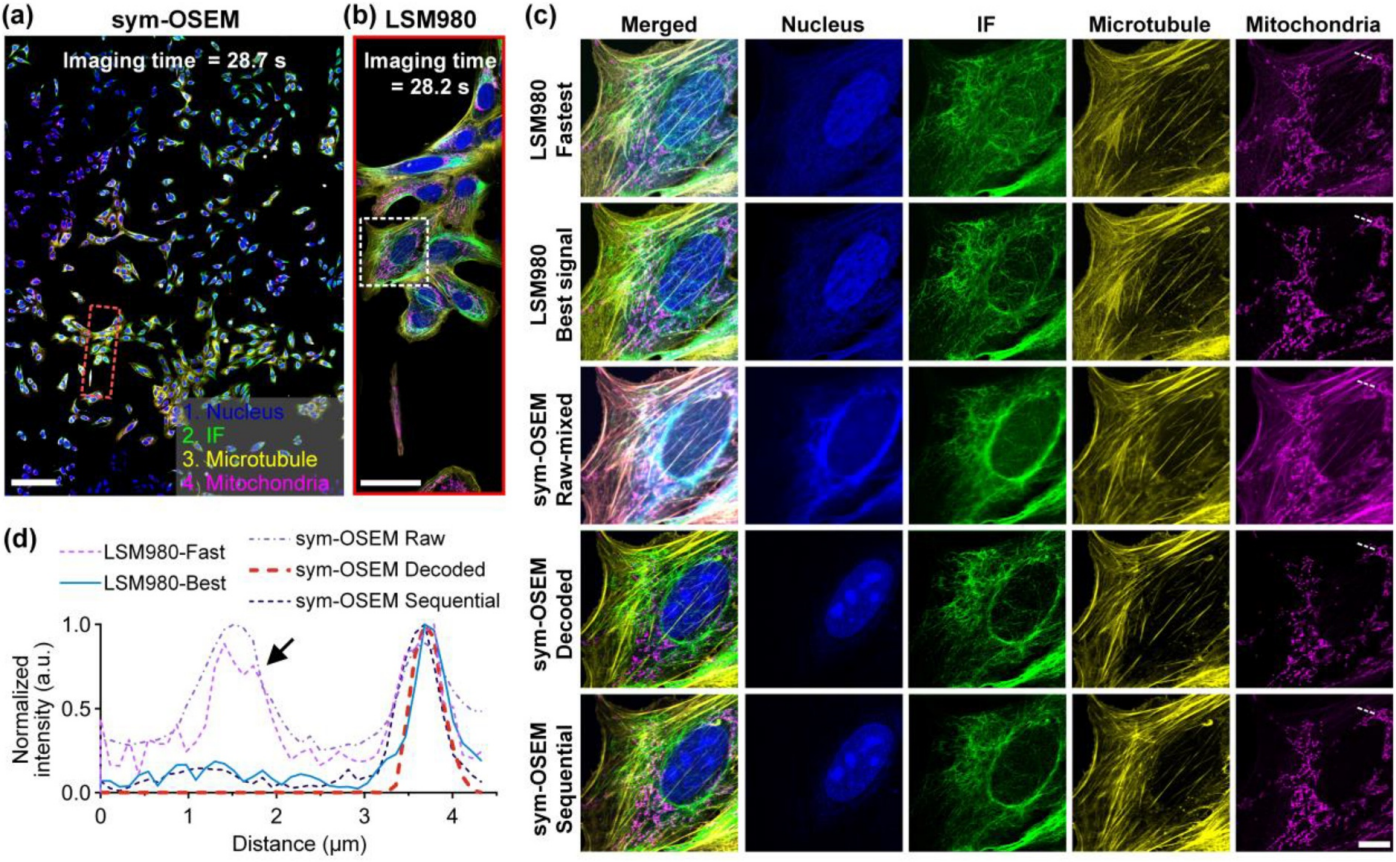
Four‐color imaging of U2OS cells. (a) Multiplexed fluorescence imaging of a U2OS cell smear, showing four fluorescent signals: Nuclei (blue), intermediate filaments (green), microtubules (yellow), and mitochondria (magenta). The image was acquired using sym‐OSEM and took 28.7 s. (b) Four‐color imaging of the boxed region in panel (a) using the commercial Zeiss LSM980 microscope with an acquisition time of 28.2 s. (c) Enlarged views of the boxed region in panel (b). From top to bottom: imaging results from the LSM980 Fastest mode and Best signal mode, and the sym‐OSEM raw‐mixed, differentially decoded, and sequential reference images. (d) Signal intensity profiles along the dashed lines in panel (c). Scale bars: 200 μm in panel (a), 50 μm in panel (b), 10 μm in panel (c).

Figure 5c compares the LSM980 Fastest and Best Signal modes with the raw‐mixed, differential decoded, and sequential reference images from sym‐OSEM. The LSM980 Fastest mode and sym‐OSEM raw‐mixed measurements retained visible interchannel leakage. Sequential LSM980 acquisition in Best Signal mode reduced leakage at the cost of throughput. Differential decoding separated all four labels and reproduced the principal structures visible in the sequential reference images.

Intensity profiles through the mitochondrial channel show leakage from the microtubule channel (Figure 5d). This peak was visible in the LSM980 Fastest and sym‐OSEM raw‐mixed images but was suppressed in the LSM980 Best Signal and differentially decoded images. Differential decoding also reduced background relative to the raw measurement. These comparisons indicate that sym‐OSEM can combine higher area throughput with multicolor separation and optical sectioning under the evaluated conditions.

### 3.5 Simultaneous Six‐Color Imaging of Lymph Node Tissue

To test extension beyond four colors, we performed simultaneous six‐color imaging of a mouse lymph node section and compared differential decoding with PSF‐based linear decoding, deep‐learning decoding, sequential reference imaging, and LSM980 acquisition.

We obtained samples from mouse inguinal lymph nodes. We fixed fresh tissue in 4% paraformaldehyde for 12 h, dehydrated it in 30% sucrose at 4°C overnight, embedded it in optimal cutting temperature (OCT), cryosectioned it at 8 μm thickness, and immunostained it. CD8^+^ T cells (cyan) and natural killer (NK) cells (yellow) were labeled by endogenous GFP and tdTomato, respectively. The other four cell types were immunolabeled. CD3^+^ T cells (blue) were labeled with CD3^+^ T‐Brilliant Violet 421 (100336, ThermoFisher). Stromal cells (green) were labeled with anti‐vimentin primary antibody and Alexa Fluor 514 secondary antibody (A‐31558, ThermoFisher). B cells (purple) were labeled with CD45R/B220‐Alexa Fluor 594 (103254, BioLegend). Dendritic cells (DCs) (red) were labeled with DC‐Alexa Fluor 647 (117312, BioLegend).

To acquire six‐color off‐axis spectrally encoded images, four lasers (405, 488, 561, and 633 nm) simultaneously excited the six labels, and we collected fluorescence through a 20×, 1.0‐NA objective. Two dichroic mirrors split the emission among three parallel cameras, each recording two symmetrically encoded channels (Figure 6a). Sequential excitation of each condition provided reference images. Residual leakage was estimated from single‐label regions and removed by scaled subtraction to yield crosstalk‐corrected sequential references. Ideally, we would validate this correction with single‐label tissue controls. However, such controls were not technically reliable for this specimen, so we estimated the correction empirically from label‐dominant regions within the same specimen. The crosstalk‐corrected sequential images are comparison references rather than independently validated ground truth.

**FIGURE 6:**
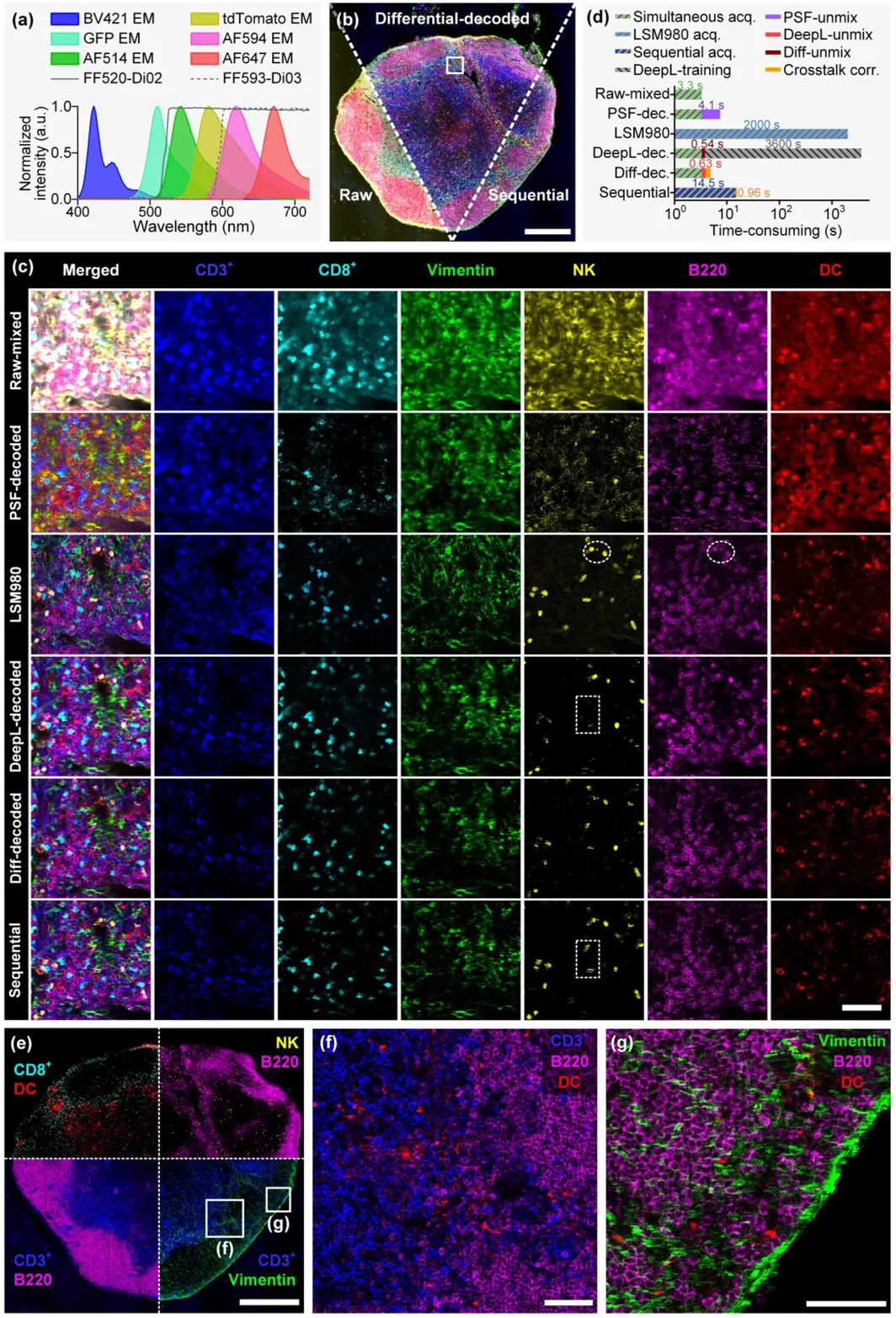
Six‐color imaging of mouse lymph node tissue. (a) Emission spectra of the six‐color fluorescent labels in the sample and transmission spectra of the dichroic mirrors in the detection path. (b) Six‐color multiplexed fluorescence imaging of lymph node tissue, with raw‐mixed, differentially decoded, and sequential reference images stitched together to form a complete view of the sample. (c) Enlarged views of the boxed region in panel (b). From top to bottom: Raw‐mixed, PSF‐based linear decoded, LSM980, deep‐learning decoded, differentially decoded, and sequential reference images. From left to right: Merged six‐color images and monochromatic images of each channel. (d) Comparison of imaging strategies by time. (e) Spatial distribution of each fluorescence signal; the four quadrant regions show different fluorescence combinations. (f) T‐B border region of the lymph node. (g) Subcapsular sinus region of the lymph node. Scale bars: 0.5 mm in panels (b) and (e); 50 μm in panels (c), (f), and (g).

For comparison with our previous OSEM method, we processed the raw‐mixed measurements using PSF‐based linear decoding, deep‐learning decoding, and differential decoding. PSF‐based decoding used simulated PSFs to unmix the raw‐mixed sym‐OSEM images. Deep‐learning decoding trained the previously reported ResUNet on one‐fifth of the imaged area using paired 512 × 512‐pixel patches from the raw‐mixed and sequential reference images, and applied it to the remaining area. Differential decoding separates components encoded by different excitation lines within each camera. Separating fluorophores that share an excitation wavelength, such as GFP/Alexa Fluor 514 or tdTomato/Alexa Fluor 594, also relies on dichroic emission‐band partitioning across the three cameras and subsequent crosstalk correction. Residual leakage in the differential outputs is reduced using a matrix estimated from label‐dominant regions of the same specimen (see Supporting Information S1: Table S3). Matrix accuracy may influence weak signals, relative intensities, and apparent colocalization, particularly for larger off‐diagonal coefficients. The resulting six‐color separation reflects combined contributions of the complete workflow. We also imaged the same section with the LSM980 in Best Signal mode using a 20×, 0.8‐NA objective. We also required crosstalk correction for the LSM980 images; we extracted reference spectra from single‐label regions and performed spectral unmixing in ZEN (Zeiss microscope software) using its built‐in functions.

Figure 6b compares the raw‐mixed, differential decoded, and sequential reference images from sym‐OSEM. Enlarged views in Figure 6c additionally show PSF‐based linear decoding, LSM980, and deep‐learning results. Raw‐mixed measurements contained substantial interchannel leakage. PSF decoding still exhibited substantial residual crosstalk because the simulated reference PSFs were insufficiently accurate. LSM980 images also showed slight leakage between selected labels, for example, NK‐cell signal bleeding into the B‐cell channel, due to cross‐excitation and spectral overlap. Deep‐learning decoding recovered the overall channel distributions but omitted localized NK‐cell signal in the highlighted region. Differential decoding more closely reproduced the reference distributions across the six displayed channels.

For a 2.60 mm × 2.68 mm area, sym‐OSEM acquisition took 3.3 s, sequential imaging on the same line‐scanning system took 14.5 s, and LSM980 point scanning took 33 min (Figure 6d). Post‐processing took 4.1 s for PSF linear decoding and 0.63 s for differential decoding. Differential decoding can be executed during acquisition. Deep‐learning inference took 0.54 s but required approximately 1 h for training‐data acquisition and network training. Crosstalk correction took approximately 0.96 s for sym‐OSEM and 2.1 s for LSM980. Among the tested workflows, sym‐OSEM provided the shortest combined acquisition and processing time.

The six recovered channels delineated complementary lymph node compartments (Figure 6e). CD3‐positive T cells were concentrated in the paracortex, CD8‐positive T cells were enriched in the deep paracortex, and B220‐positive B cells formed cortical follicles adjacent to the T‐cell zone. Vimentin‐positive stromal structures formed a reticular network, whereas the NK‐cell and dendritic‐cell labels were sparser in the paracortical and subcapsular regions. Enlarged views show the T–B border (Figure 6f) and subcapsular region (Figure 6g), two anatomical contexts in which simultaneous visualization of multiple labels may facilitate spatial analysis [22, 23, 24]. These results demonstrate the feasibility of sym‐OSEM for simultaneous six‐color imaging in a tissue section.

## 4 Discussion

We developed sym‐OSEM to combine simultaneous multicolor acquisition, optical sectioning, and differential decoding within an off‐axis line‐scanning architecture. Its defining feature is the paired encoding geometry, where one spectral component is matched across symmetric subdetectors, while the adjacent component is differentially modulated. Direct subtraction can therefore suppress the matched spectral contribution and common‐mode background without specimen‐specific PSF, reference spectrum, or trained model.

The experiments support three main conclusions. First, symmetric encoding reduced dependence on prior‐based unmixing across the tested specimens. Second, parallel excitation and detection preserved acquisition speed from two to six demonstrated channels, although scaling required additional cameras and spectral partitions. Third, differential decoding combined channel separation with suppression of out‐of‐focus background. In the four‐color experiment, sym‐OSEM achieved 59‐fold greater area throughput than the confocal microscope under tested acquisition settings. The serial brain‐volume and lymph‐node experiments further showed compatibility with large‐area and multiplexed tissue imaging.

The six‐color lymph node experiment demonstrates scalability of the three‐camera implementation rather than unrestricted independence from channel count. Additional channels would require sufficient excitation selectivity, detector capacity, spectral partitioning, and photon budget. Within the demonstrated configuration, simultaneous acquisition avoided the temporal offsets associated with sequential multicolor imaging and preserved the spatial correspondence of the six labels.

Sym‐OSEM requires one‐time optical alignment to position each excitation beam at the designated mid‐seam locations, with no re‐alignment needed across samples. In practice, strong intensity imbalance can degrade weak‐channel recovery fidelity through residual crosstalk and shot noise from the stronger component. Fluorophore selection or laser‐power adjustment can help balance the detected signals. Extending sym‐OSEM to live‐cell imaging would open new opportunities; the line‐scanning geometry and low phototoxicity are promising in this regard.

In summary, symmetric off‐axis encoding enables deterministic differential separation of simultaneously acquired fluorescence channels while suppressing common‐mode background. More broadly, this work demonstrates that strategic optical encoding can simplify component separation, offering a practical strategy for high‐throughput multicolor microscopy.

## Author Contributions

**Jiangjiang Zhao:** conceptualization, methodology, investigation, formal analysis, writing – review and editing, writing – original draft, data curation. **Zhangheng Ding:** conceptualization, methodology. **Zhan Zhang:** visualization, software. **Jie Zhang:** investigation. **Shuhong Qi:** investigation. **Jing Yuan:** conceptualization, methodology, data curation, investigation, formal analysis, supervision, funding acquisition, project administration, resources, writing – review and editing.

## Funding

This work was supported by the National Natural Science Foundation of China (Grants 62325502, 82527805, 81827901) and Taihu Lake Innovation Fund for Future Technology (HUST: 2025‐A‐2).

## Ethics Statement

The Institutional Animal Care and Use Committee of Huazhong University of Science and Technology approved all animal procedures (approval No. 3139).

## Supporting information


Supporting Information S1

## Data Availability

The data supporting the findings of this study are available from the corresponding author upon reasonable request.
