# Supplementary material for: Symmetric Off‐Axis Spectral Encoding Enables High‐Throughput Six‐Color Optical Sectioning Imaging
Source: Nanophotonics. 2026 Nov 7;15(21):e70367. doi: 10.1002/nap2.70367 (PMC13744097; doi:10.1002/nap2.70367)
Supplement: Supplementary file 1 [file NAP2-15-e70367-s001.docx]

**Supplementary Information for**

**Symmetric off-axis spectral encoding enables high-throughput six-color optical sectioning imaging**

Jiangjiang Zhao, Zhangheng Ding, Zhan Zhang, Jie Zhang, Shuhong Qi, Jing Yuan*

MoE Key Laboratory for Biomedical Photonics, Wuhan National Laboratory for Optoelectronics, Huazhong University of Science and Technology, Wuhan 430074, China

*Corresponding author: yuanj@hust.edu.cn

Section 1: Simulation analysis of error sensitivity in differential decoding

Section 2: Axial Sectioning Response Measurement

Section 3: Three-Dimensional Spatial Response Measurement

Section 4: Scan-coordinate registration and weighted differential forward model

Figure S1. Simulation-based sensitivity analysis of differential decoding

Figure S2. Fluorophore intensity ratio versus decoded SNR

Figure S3. Fluorescent structures at different axial positions within the same field of view

Figure S4. Axial-sectioning measurement using a fluorescent cross-matrix calibration slide

Figure S5. Three-dimensional bead-response measurements

Table S1. Joint-error analysis

Table S2. Imaging parameters for the four-color U2OS-cell comparison

Table S3. Crosstalk matrix for the six-color differential-decoded images

**Section 1:** **Simulation analysis of error sensitivity in differential decoding**

We performed a simulation-based sensitivity analysis using the same dual-color three-dimensional virtual sample as in the simulation validation in Section 3.1 of the manuscript. We varied one error term at a time while keeping the remaining parameters at their nominal values. The tested error terms included line-illumination spot drift, misregistration between subdetector images, gain imbalance between symmetric subdetectors, signal photon budget, spherical aberration, defocus, and channel-intensity imbalance. For each condition, we calculated the crosstalk increase, the broadening of the axial-sectioning response, and the peak SNR of the decoded image.

**Figure S1.** Simulation-based sensitivity analysis of differential decoding. (a) Line-illumination spot drift. (b) Misregistration between subdetector images. (c) Gain imbalance between symmetric subdetectors. (d) Signal photon budget. (e) Spherical aberration. (f) Defocus. Dashed lines indicate the 10-pp extra-crosstalk, 2-fold axial-broadening, and 20-dB peak-SNR tolerance limits.

Line-illumination spot drift and image misregistration mainly increase residual crosstalk because they disrupt the matching of the unwanted channel across the symmetric sub-detectors. Gain mismatch introduces an amplitude imbalance between the paired measurements and can also increase crosstalk. Detector noise is characterized by the signal photon budget (i.e., the number of electrons corresponding to the brightest pixel in the raw mixed image). It primarily affects the SNR and does not introduce crosstalk. Spherical aberration is symmetric about the optical axis and even in the radial coordinate, so it does not break the mirror symmetry underlying differential subtraction. Its crosstalk tolerance is therefore relatively loose. Nevertheless, it still broadens the PSF and degrades optical sectioning. Defocus only shifts the axial working point and has little effect on decoding performance; within ± 5 µm, the crosstalk increase remains below 7.4%, and the SNR remains above 32 dB.

The tolerance thresholds for the three metrics were set empirically to a crosstalk increase of <10%, FWHM broadening of <2-fold, and peak SNR of >20 dB, and are marked by dashed lines in the sub-panels. A joint-error scenario in which the major error terms were simultaneously set to half tolerance values is summarized in Supplementary Table S1.

**Table S1. Joint-error analysis**

| **Error scenario** | **Crosstalk increase (%)** | **FWHM broadening** | **Peak SNR (dB)** |
| --- | --- | --- | --- |
| All errors at half tolerance | 7.5 | 1.1× | 26.1 |
| excluding line-illumination spot drift | 6.0 | 1.1× | 26.6 |
| excluding image misregistration | 4.7 | 1.1× | 26.0 |
| excluding subdetector gain imbalance | 5.5 | 1.1× | 26.0 |
| excluding detector noise  (reference photon budget, 10000 e⁻) | 7.5 | 1.1× | 29.2 |
| excluding spherical aberration | 6.4 | 1.0× | 27.3 |
| excluding defocus distance | 7.0 | 1.1× | 26.2 |
| excluding channel power imbalance | 7.5 | 1.1× | 29.0 |

All major error terms were simultaneously set to half of their tolerance values. Removing one error term at a time from the joint-error scenario shows that the SNR is mainly limited by detector noise and channel-intensity imbalance, crosstalk is mainly affected by line-spot drift and image misregistration, and FWHM broadening is mainly affected by aberration.

**Figure S2.** Fluorophore intensity ratio versus decoded SNR.

We also simulated how fluorescence intensity imbalance affects differential decoding. Intensity imbalance does not break the symmetry underlying differential subtraction and thus does not increase nominal crosstalk. However, because subtraction removes only the mean crosstalk, not its shot noise, a strong signal can still degrade the effective SNR of weak channels. Both decoded channels remain above the adopted 20-dB tolerance throughout the displayed range of 0.2–4. The plotted 25-dB line is an additional reference level, crossed by the weaker channel near 0.30.

**Section 2:** **Axial Sectioning Response Measurement**

The axial sectioning response was measured using a fluorescent calibration slide (Argo-SIM-SLG034, Argolight) with a 60× water-immersion objective (LUMFLN60XW, NA 1.1, Olympus) under 488-nm excitation. A z-stack containing 200 planes was acquired with a 0.1-µm axial step. During acquisition, the sCMOS camera was operated in the 16-row subarray mode, so that images from different off-axis subdetectors were recorded independently. Summing the 16 row images generated an approximate wide-field image. The central row conjugated to the line-illumination beam was used as the line-confocal image. Subtracting an edge-row image from the central-row image generated the differential image for this single-channel measurement.

**Figure S3.** Fluorescent structures at different axial positions within the same field of view. (a) Approximate wide-field, line-confocal, and differential images of the fluorescent cross-matrix pattern. The horizontal lines are located at different axial positions. (b) Intensity profiles along the dashed lines in (a). Scale bar: 10 µm.

In the differential image at the reference focal plane, the in-focus vertical lines remain visible, whereas out-of-focus horizontal-line signals are strongly suppressed. At ΔZ = +1.5 µm, the trend is reversed, with the vertical-line signals reduced and the horizontal-line signals near that axial plane becoming stronger. The wide-field image retains substantial out-of-focus signal, whereas the line-confocal image shows an intermediate response.

**Figure S4.** Axial-sectioning measurement using a fluorescent cross-matrix calibration slide. (a) Approximate wide-field, line-confocal, and differential images at the reference plane and at ΔZ = +1.5 µm. The vertical lines are located in a common reference plane, whereas the horizontal lines are located at different axial positions. (b) Representative normalized axial-response curves and corresponding FWHM values. (c) Distribution of FWHM values obtained from 16 horizontal-line structures. Data are shown as mean ± SD. Scale bar: 10 µm.

The cross-matrix pattern of the calibration slide contains 4 × 4 cross structures. The vertical lines are located in a common reference plane, whereas the horizontal lines are positioned at different axial planes with a nominal step of 0.1 µm over a range of 0 – 1.5 µm. Figure S3 shows the wide-field, line-confocal, and differential images acquired at the same axial plane, together with line profiles through structures at different axial positions. In Figure S3a, the signal from the defocused horizontal line at the lower-right corner is strongly suppressed in the differential image and partially rejected by the slit in the line-confocal image, whereas it remains intense in the wide-field image. Figure S3b shows line profiles along a dashed line crossing four horizontal structures with progressively increasing defocus. In the wide-field profile, the four corresponding peaks have comparable intensities, indicating almost no optical sectioning. In the line-confocal profile, defocused signals are partially rejected, but even the most defocused line retains a noticeable residual. The differential image provides better sectioning and stronger suppression of defocused signals.

We quantified the axial response by measuring the background-subtracted integrated intensity of each horizontal line over the z stack. Each horizontal line was analyzed with respect to its own focus plane. The axial-response FWHM values were 3.74 ± 0.06 µm for the wide-field image, 2.55 ± 0.05 µm for the line-confocal image, and 1.84 ± 0.05 µm for the differential image (mean ± SD, n = 16 line structures). Thus, under this measurement condition, differential processing narrowed the axial-response width by approximately 50.8% relative to the approximate wide-field image and by approximately 27.8% relative to the line-confocal image.

**Section 3:** **Three-Dimensional Spatial Response Measurement**

Fluorescent beads with a diameter of 200 nm (T14792, Thermo Fisher) were imaged in three excitation/emission conditions: 488/515 nm, 561/580 nm, and 633/680 nm. The imaging was performed with a 60× water-immersion objective (LUMFLN60XW, NA 1.1, Olympus), with a lateral sampling interval of 108 nm and an axial step of 100 nm. The sCMOS camera was operated in the 16-row subarray mode. Approximate wide-field, line-confocal, and differential-decoded images were reconstructed from the same raw data, following the reconstruction procedure described in Supporting Information Section 2.

For each fluorescence channel, 15 beads with adequate signal intensity were randomly selected, and three-dimensional response widths were measured along X, Y, and Z. The X direction is perpendicular to the illumination line, the Y direction is along the line, and Z is the axial direction. These are apparent FWHM values that include bead-induced broadening. The deconvolved system PSF would be narrower. We therefore refer to them as bead-response FWHM values rather than intrinsic PSF widths.

The results show that the line-confocal and differential images substantially narrow the X and Z responses relative to the approximate wide-field image, while the Y response changes little. This is consistent with the line-scanning geometry, in which confocal sharpening and differential suppression act mainly perpendicular to the illumination line and along the axial direction. Since the differential image is formed by subtracting adjacent subdetector images, it further compresses the X-direction PSF main lobe and cancels uniform out-of-focus background relative to line-confocal imaging, thereby narrowing the X and Z responses further.

**Figure S5.** Three-dimensional bead-response measurements. Representative X, Y, and Z profiles of 200-nm fluorescent beads measured at 488/515 nm, 561/580 nm, and 633/680 nm. Dots are measured data, and curves are fits. Panels show statistics from 15 randomly selected beads (mean ± SD, n = 15). Green, orange, and blue text indicate wide-field, line-confocal, and differential-decoded images, respectively.

**Section 4: Scan-coordinate registration and weighted differential forward model**

**4.1 Scanning coordinates and effective response**

We first derive the response for one camera and one fluorescence component q. All lateral positions are expressed in sample-plane units after magnification and orientation mapping. Let s(t) be the instrument origin in specimen coordinates during scanning, eq the excitation-line position, and di the center of detector row i relative to that origin. The corresponding line and row positions in the specimen are s(t)+eq and s(t)+di. The scan position is assumed known so that different acquisition times can be assembled in a common specimen coordinate system. Fluorescence is assumed linear and stationary during the relevant row acquisitions. Within the modeled field, illumination is locally uniform along the line direction, and the response is approximately shift invariant. Equal exposure/scan-blur conditions are assumed; otherwise the exposure integration must be included in the effective kernel. These assumptions do not imply equality of different row responses.

Let Lq be the excitation intensity response and Dq the emission response integrated over a detector-pixel aperture. A row factor gi includes its relative detector gain. The contribution of component q to the conditional mean raw row measurement is

$$\bar{Y}_{i,q}(s,y₀,z₀) = P_{q}\iiint X_{q}(x,y,z)G_{i,q}(x-s-dᵢ,y-y₀,z-z₀) dx dy dz (S1)$$

$$G_{i,q}(\xi,\eta,\zeta) = g_{i}L_{q}(\xi+dᵢ-e\_q,\zeta)D_{q}(\xi,\eta,\zeta) (S2)$$

Dq includes finite pixel-aperture integration, rather than point detection. Pq is a common component intensity scale. The relative row collection amplitudes remain in Gi,q; normalizing each row independently would remove part of the encoding and is not implied by this definition. For a pixel aperture Aq and an object-space emission PSF h_em,q, the detection response can be written as

$$D_{q}(\xi,\eta,\zeta) = \int\int A_{q}(\rhoₓ,\rhoᵧ)h_{em,q}(\rhoₓ-\xi,\rhoᵧ-\eta,\zeta) d\rhoₓ d\rhoᵧ (S3)$$

Scan-coordinate assembly associates detector row i with the physical sample position u=s+di. For a single component, write Ji,q(u,y₀,z₀)=Yi,q(u−di,y₀,z₀). Its conditional mean then becomes

$$\bar{J}_{i,q}(u,y₀,z₀) = P_{q}\iiint X_{q}(u+\xi,y₀+\eta,z₀+\zeta)G_{i,q}(\xi,\eta,\zeta) d\xi d\eta d\zeta(S4)$$

To use conventional convolution notation, define Hi,q(r)=Gi,q(−r), where r=(ξ,η,ζ). For two components, the assembled mixed image evaluated at r₀=(u,y₀,z₀) satisfies

$$I_{i} = \sum_{q=1}^{2} P_{q}H_{i,q} * X_{q} + B_{i} + \eta_{i} (S5)$$

The convolution is three-dimensional and evaluated at the output plane. Bi describes deterministic instrumental offsets. Out-of-focus fluorescence is included in the three-dimensional convolution and is not reclassified as an additive instrumental offset. ηi is conditionally zero-mean shot/read noise.

**4.2 Fixed registration and the mirror symmetry**

The actual workflow additionally applies a subpixel registration operator Ti calibrated once on the instrument and held fixed across specimens. For an ideal continuous translation, (Ti f)(r)=f(r+τi eₓ). In sampled data, Ti denotes the actual fixed interpolation operator; the same operator must be applied to simulated or measured kernels when evaluating mismatch. The registered measurement and kernel are

$$\tilde{I}_{i} = T_{i}I_{i}, \tilde{H}_{i,q} = T_{i}H_{i,q} (S6)$$

Before registration, symmetric offsets da−eq=+a and db−eq=−a imply the following mirror relation if illumination and detection are even in the scan direction and row gains are matched:

$$H_{b,q}(\xi,\eta,\zeta) = H_{a,q}(-\xi,\eta,\zeta) (S7)$$

The relation is a geometric symmetry, not pointwise equality. Translation can align displaced main lobes, but need not align different shapes or maintain alignment through all contributing depths. Define the registered pair mismatch as

$$\varepsilon_{ab,q} = \tilde{H}_{a,q} - \tilde{H}_{b,q} (S8)$$

For arbitrary fluorophore distributions, exact mean cancellation by this pair requires εab,q≡0. This condition is also necessary in the shift-invariant model: a point-like nonnegative fluorophore distribution probes the difference kernel directly. Matching integrated intensities or centroid positions alone does not establish this equality.

A local scalar-modulation approximation explains approximate matching. If Lq varies little over the main support of Dq, then Gi,q(ξ,η,ζ)≈gi Lq(ai,ζ)Dq(ξ,η,ζ), with ai=di−eq. Matched gains and Lq(a,ζ)=Lq(−a,ζ) give equal responses within that approximation. Retaining the illumination variation gives, before additional registration,

$${\Delta G}_{q}(\xi,\eta,\zeta) = g D_{q}(\xi,\eta,\zeta)[L_{q}(\xi+a,\zeta) - L_{q}(\xi-a,\zeta)] (S9)$$

$${\Delta G}_{q}(\xi,\eta,\zeta) = 2g\xi{\partialₓL}_{q}(a,\zeta)D_{q}(\xi,\eta,\zeta) + O(\xi^{3}) (S10)$$

Under the even-response assumptions, ΔGq is odd in ξ, and its ξ integral is zero. This cancels a component uniform in the scan direction, but its response to an arbitrary structured specimen is generally nonzero. Airy sidelobes and symmetric pixel integration do not by themselves invalidate mirror symmetry; they can prevent the two mirror-related kernels from being identical after a fixed translation. Oddness and zero integrated response are therefore distinct from cancellation for arbitrary objects.

A separable Gaussian example makes the depth limitation explicit. Assume Lq(ξ,ζ)∝exp[−ξ²/(2σe²(ζ))] and Dq(ξ,η,ζ)∝F(η,ζ)exp[−ξ²/(2σd²(ζ))]. For matched gains and symmetric offsets, completing the square gives

$$G_{\pm,q}(\xi,\eta,\zeta) = C(a,\zeta)F(\eta,\zeta) exp[\frac{-(\xi\pm a\beta(\zeta))^{2}}{2\sigma^{2}(\zeta)}] (S11)$$

$$\beta(\zeta) = \frac{\sigma_{d}^{2}(\zeta)}{\sigma_{e}^{2}(\zeta)+\sigma_{d}^{2}(\zeta)}, \sigma^{2}(\zeta) = \frac{\sigma_{e}^{2}(\zeta)\sigma_{d}^{2}(\zeta)}{\sigma_{e}^{2}(\zeta)+\sigma_{d}^{2}(\zeta)} (S12)$$

At a fixed depth, these Gaussian kernels have equal amplitudes and shapes but shifted centers. Aligning them at ζ=0 with a fixed translation leaves the following relative center displacement at another depth:

$$|\Delta x(\zeta)| = 2|a| |\beta(\zeta) - \beta(0)| (S13)$$

For nonzero a, a single translation aligns this Gaussian pair throughout depth only if β(ζ) is constant over that range.

**4.3 Weighted differential decoding and residual component kernels**

Let i=1,…,N index the detector-row measurements included in decoding. The fixed weights wℓi specify the differential combination for output ℓ and are chosen according to the calibrated illumination–detector geometry. For the two-component example, CH1 retains component 1, and CH2 retains component 2. Differential rejection of a contribution common across registered rows uses the zero-sum condition

$$W = [w_{\mathcal{l}i}], \mathcal{l} = 1,2, i = 1,\ldots,N; \sum_{i=1}^{N} w_{\mathcal{l}i} = 0 (S14)$$

$$D_{\mathcal{l}} = \sum_{i=1}^{N} w_{\mathcal{l}i}\tilde{I}_{i}, K_{\mathcal{l}q} = \sum_{i=1}^{N} w_{\mathcal{l}i}\tilde{H}_{i,q} (S15)$$

The first weight vector suppresses component q=2 while retaining q=1, and the second suppresses q=1 while retaining q=2. An elementary paired difference uses opposite weights on two registered rows. More generally, the unwanted-component kernels are the complete weighted residuals:

$$\varepsilon_{1} = K_{12} = \sum_{i=1}^{N} w_{1i}\tilde{H}_{i,2} (S16)$$

$$\varepsilon_{2} = K_{21} = \sum_{i=1}^{N} w_{2i}\tilde{H}_{i,1} (S17)$$

$$D_{1} = P_{1}K_{11} * X_{1} + P_{2}\varepsilon_{1} * X_{2} + {\Delta B}_{1} + {\Delta\eta}_{1} (S18)$$

$$D_{2} = P_{2}K_{22} * X_{2} + P_{1}\varepsilon_{2} * X_{1} + {\Delta B}_{2} + {\Delta\eta}_{2} (S19)$$

Here ΔBℓ=Σi wℓi TiBi and Δηℓ=Σi wℓi Tiηi. Exact mean separation for arbitrary specimens requires ε₁≡ε₂≡0 and balanced additive offsets. Pairwise equality is sufficient, but not necessary: individual pair residuals can partly compensate in the combined weighted kernel. Such compensation must be evaluated at kernel level, rather than inferred from a particular specimen. Nonzero K₁₁ and K₂₂ retain spatially filtered target signals; the outputs are not assumed to be unfiltered fluorophore distributions. Because each weight vector sums to zero, a contribution identical across the registered row measurements cancels. Out-of-focus suppression is approximate when those row contributions are only approximately common.

**4.4 Six fluorophores, four excitation lines, and three cameras**

For the six-fluorophore experiment, let j=1,…,6 index BV421, EGFP, AF514, tdTomato, AF594, and AF647; k=1,…,4 index the 405-, 488-, 561-, and 633-nm excitation lines; and m=1,…,3 index the cameras. The full scan-assembled measurement retains all excitation and detection paths:

$$I_{m,i} = \sum_{j=1}^{6} \sum_{k=1}^{4} P_{j,m,k}(H_{m,i,j,k} * X_{j}) + B_{m,i} + \eta_{m,i} (S20)$$

Pj,m,k includes the common intensity scale, excitation efficiency, spectral transmission, and detection efficiency for that fluorophore/path. Hm, i,j,k retains the relative detector-row collection amplitude and spatial shape, including excitation-line position and emission-band response. Individual row kernels are not separately normalized to equal integrals. The registration operator Tm,i is fixed for the instrument. Each output ℓ selects camera m(ℓ) and the appropriate differential weight vector according to that camera’s calibrated line ordering:

$$D_{\mathcal{l}} = \sum_{i=1}^{N} w_{\mathcal{l}i}T_{m(\mathcal{l}),i}I_{m(\mathcal{l}),i} (S21)$$

$$\mathcal{K}_{\mathcal{l}j} = \sum_{k=1}^{4} P_{j,m(\mathcal{l}),k}(\sum_{i=1}^{N} w_{\mathcal{l}i}T_{m(\mathcal{l}),i}H_{m(\mathcal{l}),i,j,k}) (S22)$$

$$D_{\mathcal{l}} = \sum_{j=1}^{6} \mathcal{K}_{\mathcal{l}j} * X_{j} + {\Delta B}_{\mathcal{l}} + {\Delta\eta}_{\mathcal{l}} (S23)$$

The six outputs are ordered as CH1=Cam1–405, CH2=Cam1–488, CH3=Cam2–488, CH4=Cam2–561, CH5=Cam3–561, and CH6=Cam3–633, as in Table S3. These labels identify the intended excitation-associated output, not a mathematically isolated fluorophore. Components sharing an excitation line cannot be distinguished by that excitation position alone. EGFP/AF514 and tdTomato/AF594 additionally rely on camera spectral partitioning and residual crosstalk correction.

$$d(r) \approx A s(r) + \varepsilon(r) (S24)$$

This final expression is the empirical six-channel mixing approximation used for residual correction. d is the vector of differential outputs, s contains separated spatially filtered channel signals rather than the original Xj, and A is the effective matrix in Table S3, with rows corresponding to outputs and columns to fluorophores. Its diagonal normalization does not imply independent normalization of the optical row kernels. ε contains noise, offsets, and model mismatch. A was estimated from label-dominant regions. "Reference-free" applies to the differential operation after instrument calibration and excludes this specimen-derived matrix correction.

**Table S2. Imaging parameters for the four-color U2OS-cell comparison**

| **Imaging parameter** | **Sym-OSEM** | **Zeiss LSM980 (Best Signal)** |
| --- | --- | --- |
| Objective NA | 1.1 | 1.4 |
| Objective magnification | 60× | 63× |
| Pixel size | 0.108 µm × 0.108 µm | 0.071 µm × 0.071 µm |
| Bit depth | 16 bit | 8 bit |
| Pixel time | – | 0.28 µs |
| Frame time | 0.16 ms (1 × 2048 pixels) | 2.35 s (1908 × 1908 pixels) |
| Imaging range (pixels) | 13687 × 19000 | 1908 × 5321 |
| Imaging range (area) | 1.483 mm × 2.052 mm | 0.135 mm × 0.378 mm |
| Total imaging time | 28.7 s | 28.2 s |
| Imaging speed | 6.362 mm² min⁻¹ | 0.109 mm² min⁻¹ |

**Table S3. Crosstalk matrix for the six-color differential-decoded images**

|  | **BV421** | **EGFP** | **AF514** | **tdTomato** | **AF594** | **AF647** |
| --- | --- | --- | --- | --- | --- | --- |
| CH1  (Cam1-405) | 1.00 | 0.15 | 0.02 | 0.08 | 0.05 | 0.07 |
| CH2  (Cam1-488) | 0.00 | 1.00 | 0.03 | 0.00 | 0.00 | 0.00 |
| CH3  (Cam2-488) | 0.00 | 0.57 | 1.00 | 0.26 | 0.11 | 0.00 |
| CH4  (Cam2-561) | 0.00 | 0.00 | 0.03 | 1.00 | 0.06 | 0.05 |
| CH5  (Cam3-561) | 0.00 | 0.00 | 0.00 | 0.65 | 1.00 | 0.13 |
| CH6  (Cam3-633) | 0.00 | 0.00 | 0.07 | 0.00 | 0.14 | 1.00 |
